# Some Remarks on the Employment of Chloroform by Dentists

**Published:** 1856-01

**Authors:** 


### ARTICLE IV.

### *Some Remarks on the Employment of Chloroform by Dentists.*

ONE of the most important questions which can be propounded to any practitioner of surgery, whether general or special, is that of the propriety of employing chloroform in any given case. It is a question, too, which cannot be evaded, for even if the operator himself be indisposed to administer it, and refrain entirely from suggesting its employment, yet the patient will be very likely to propose, and may insist upon, its use. There is an instinctive shrinking from pain in all men, and many are willing to risk much in order to ensure entire immunity from suffering.

It is the purpose of this article to make some calm inquiries into the use of this agent in surgery, and especially that department of the art which is practiced by the majority of the readers of this journal. Originality is not claimed for any of the propositions advanced, the entire design of these remarks being to call attention to the present state of our knowledge in reference to this important question.

It is certainly a very remarkable fact that although this agent has been used for several years, in a very large number of cases of every age, of both sexes, and for almost every variety of disease, and although there have been many deaths occurring under its influence, yet we are still far from a rational and satisfactory account of the method in which it kills. First the brain, then the lungs, and last of all, the heart have been suspected. Ricord recommended insufflation. This was found to be difficult in some cases, and it was believed that the trouble arose from the position of the epiglottis covering the orifice of the trachea. It became fashionable then to thrust the finger into the mouth of the pulseless patient, to lift up the epiglottis and then vigorously to inflate the lungs. All reported cases under this mode of treatment had a favorable termination, but as the journals have gradually become silent upon the subject, it is fair to infer that the theory and practice have been both laid upon the shelf together.

Of late, the favorite theory has been that which attributes the fatality attendant upon the use of this agent to a disease of the heart, in which the muscular fibres of that organ are converted into fat. The only known indications of the existence of this condition of the heart are a weak, intermittent pulse, liability to faint, and *arcus senilis.* This is a disease of the cornea in which the outer margin of that organ is affected with the same fatty degeneration, so that there appears to be a dull yellowish-gray ring surrounding and obscuring the outer edge of the iris. There are some grave reasons, however, for believing that this theory will share the fate of its predecessors.

In the first place, we have the undeniable fact that many of the so-called victims of chloroform have presented no evidence whatever of the presence of any such disease. The affection alluded to is peculiarly a disease of old age, and though it may come on at any period of life, it is more likely to attack the aged. It is also exceedingly difficult of diagnosis, so that the mere presence of the arcus senilis in one eye is not a certain evidence of the disease of the heart, while, upon the other hand, it may exist without any external indication of its presence. In several recent cases of death during the use of chloroform, this degeneration of the muscular fibres of the heart has been discovered, but by far the greater majority of fatal cases have exhibited no such lesion, even upon careful microscopical examination. It would be exceedingly unphilosophical to assume that the comparatively few cases in which the disease has been recognized, should be suffered to explain the many in which nothing of the kind has been detected.

Dr. Snow, in commenting upon the propriety of using chloroform in surgery, remarks, that "patients with fatty degeneration of the heart are liable to die suddenly in two distinct ways: first, with the cavities of the heart empty; and secondly, with the cavities of the heart full of blood. In the latter case, death is caused by the inability of the weakened heart to propel the blood; but, in the former case, when the patient dies by ordinary syncope, such as that occasioned by loss of blood, and where the cavities of the heart are empty, death must be caused by some undescribed condition which accompanies the disease of the heart, and not by that disease itself; for the most healthy heart would be unable to maintain the circulation when the blood no longer reached its cavities from the veins."

Now it so happens, that, in most of the recorded autopsies of patients dying suddenly under the use of chloroform, the cavities of the heart are stated to be empty. In these, then, we have the unknown element of disease supposed to accompany the cardiac affection. We have no reason, however, for inferring that this unknown quantity always requires cardiac fatty degeneration as a factor. For aught we know it may coëxist with a healthy state of the heart, and it is, in the present state of our knowledge, an unwarrantable assumption to insist upon uniting two morbid conditions between which there has not been demonstrated any necessary connection. Indeed, the chances are that the majority of those who have died under the use of this agent have had healthy hearts. Dr. Snow himself, though a warm advocate of the employment of chloroform, acknowleges this. He says:

"Out of the entire number of deaths from chloroform which are recorded, there are very few in which any considerable disease of the heart was found. In fact, the majority of those who are dead from chloroform were healthy persons in the best period of life—that is, from fifteen to thirty-five or forty, and it is most likely that they had, on an average, a sounder state of the heart than the multitude who have inhaled chloroform with impunity."

This distinguished surgeon goes still farther, and maintains that chloroform is likely to prove beneficial rather than otherwise to patients affected with this disease. He argues, that the pain attendant upon a surgical operation is likely to produce both the fatal conditions of the cavities of the heart, while chloroform, on the contrary, has a tendency to keep the circulation more equable. He says he has given chloroform with a favorable result to a great number of patients having all the symptoms of fatty degeneration of the heart. The only patient he has ever lost, while using chloroform, had this disease, but he doubts that the death was caused by the anæsthetic. He seems rather disposed to attribute it to a straining effort which the patient commenced, as if he began to feel the pain of the operation.

In speculating upon the causes of the sad results we are considering, it should always be born in mind that sudden deaths upon the operating table were not uncommon before the introduction of anæsthetics. Dr. Simpson narrates two cases in which the patients died, after the most trivial incisions. In one the skin only had been cut, and in the other, an abscess lancet had been introduced. Another case occurred in Edinburgh just before the introduction of chloroform. A patient had just had his groin shaved preparatory to the operation for strangulated hernia, and the surgeon was about to proceed, when the patient fainted and died before any incision had been made. Most of the cases of this kind may be accounted for by the effects of pain or mental emotion upon a highly susceptible nervous system. Other deaths, occurring after operations, may have depended upon syncope resulting from hemorrhage, a condition which chloroform is undoubtedly calculated to promote, as it greatly increases the tendency to bleeding.

From what has been said, it follows, that the true cause of death from chloroform remains as great a mystery as ever. The mode of death varies, the heart appearing in some cases to die first, while in others, a paralysis of respiratory motion is the first effect. Its action upon the blood is not well known. Out of the body it gives a florid, arterial hue to venous blood, and the red corpuscles are found to be dissolved, while the white globules remain unaffected. Upon the color of blood circulating in the vessels, however, it does not exert any such influence. Whether the corpuscles are dissolved or not, when this agent has been administered in the usual way, by inhalation, and whether the anæsthetic passes unchanged into the blood, are questions which do not appear to be satisfactorily answered, although the general disposition is to reply to them in the affirmative.

M. Denonvilliers has recently published, in the *Annuaire de Therapeutique*, a synopsis of the present condition of our knowledge in regard to this subject, in which he takes the same ground already assumed in this article. He says:

"An analysis of the facts shows that these serious consequences which have resulted in death, have followed the administration of chloroform in large doses for a long time, or where the dose was small and taken for a short period, or where the patients were already enfeebled, or had undergone a long or great operation; or lastly, where the subjects were young and vigorous and had borne an operation of only average magnitude and tediousness. Contrary to all expectation, these fatal results have been much more frequently observed in the last condition than in the first, so that it has been supposed that these consequences are to be attributed to some *peculiar susceptibility* of the victims, rather than to the quantity or concentration of the chloroform vapor. This susceptibility is *unknown in its nature*, and would seem to be only *temporary in its influence*, since persons have succumbed to a second chloroformization, after having borne the first operation very well."

The last remark is very important, and ought to be deeply impressed upon the mind of every one who uses this powerful remedy. It should be a sufficient caution to all not to employ so dangerous an agent in slight or unimportant operations. The mere immunity from pain is purchased at too great a risk. Dentists also should remember, that if, as M. Denonvilliers believes, "chloroform acts directly on the heart, whose contractions it can suspend immediately and indefinitely," the position in which they place their patients must necessarily favor an unfortunate result. It is true that Dr. Snow asserts, that there is no objection to a sitting posture, since chloroform usually increases the force of the circulation, and yet he strangely enough contradicts himself by recommending that the patient should be placed horizontally if symptoms of faintness come on.

Setting aside, however, the general objection of this obscure and unknown malignant power, what are the special contra-indications to its employment? Generally, it may be answered, all injuries which, by their great severity, or by the regions they affect, have a tendency to produce syncope; decided disease of the heart, brain or lungs, and all operations in which blood is likely to be poured into the air passages, or in which, either the sensations or voluntary motions of the patient may assist the surgeon.

The use of this agent once determined upon, there are certain precautions to be observed in order to ensure success. Some of these relate to the agent itself, others to the mode of administration. Denonvilliers gives the following signs by which to recognize the purity of chloroform. 1st. It gives out an agreeable odor, very much like that of the apple. 2d. Rubbed on the palm of the hand it will volatize without leaving the peculiar and nauseous odor of chlorine. 3d. One drop, when it falls in a glass of water, will sink to the bottom of the vessel, and preserve its limpidity. 4th. If it is mixed with a little sulphuric acid, no change of color is produced. All the old cumbrous apparatus having been now generally abandoned, it is only necessary to say that the handkerchief is at once the most convenient and most efficient inhaler that can be employed.

The fears of the patient are to be allayed before any chloroform is administered, or bad results may follow. Independently of this, however, fear perturbs the respiration, and it is essential that the breathing should be calm and regular. Another important point is not to administer chloroform when the stomach is full, as it may cause vomiting. This, being liable to come on at any time after the administration of the anæsthetic, may occur during the operation. It is not attended with any danger, but is a disagreeable accompaniment, and one which sometimes seriously interferes with the success of the operation. Dr. Snow says, that the best time is before breakfast, and if that is impossible, two or three hours after a slender meal.

For the rest, Denonvilliers gives such minute and satisfactory directions, that it is not necessary to do more than quote from him.

"The surgeon ought to preside over the inhalation himself. He should watch the general condition of the patient, and observe at the same time his circulation and respiration. For this purpose he should keep his finger on the radial artery, until he is ready to commence the operation, and then his place should be taken by a careful assistant, who should, from time to time, inform him of the state of the pulse and announce its variations.

"It is during the first moments of the process that chloroformization presents most dangers, and then our precautions should be greatest.

"We should begin with very small doses of chloroform and gradually increase the quantity, after having ascertained that it will be well supported. The action of chloroform being progressive, we can produce insensibility by merely continuing the inhalatives without being always compelled to repeat the dose.

"If the circulation or respiration become disturbed, we should suspend the process to permit the patient to rally, and then begin again. If the disturbance of these great functions become at all serious, it is prudent to discontinue our efforts for the time, and if possible defer the operation.

"Chloroformization should be pushed more or less far, depending on the operation which has to be performed, or the effect we desire to produce, but in all cases we should cease its administration as soon as insensibility is established.

"If it is necessary to prolong the anæsthetic state, we should return with caution to the chloroform as soon as the patient begins to recover. We can thus without pain or inconvenience to the patient, conduct an operation of an hour's duration. Nevertheless, whilst this large quantity of chloroform vapor is being absorbed, we should be on our guard against the consecutive syncope.

"Although we rarely see bad consequence supervene after the operation is concluded, yet the surgeon should not leave his patient until he has perfectly recovered his consciousness.

"In those cases in which syncope occurs, we should pursue the following course:

"1st. Place the patient on an inclined plane, so that his feet are elevated and his head occupying the lowest point.

"2d. Practice artificial respiration by regular pressure on the thoracic and abdominal walls, forcing open the mouth and drawing out the tongue, whilst we irritate the back of the throat with the finger or a spatula.

"3d. Open the windows so as to introduce a quantity of fresh and pure air.

"These means will often be successful, but only when they are carried into effect with promptness, energy and perseverance. With regard to the frictions, kneading, cold affusion, ammoniacal vapors, these are agents whose action is too uncertain and above all too slow, to be considered in any other light than as adjuvants.

"In some experiments made on animals, M. Giraudet concludes that any cause which prevents the free play of the diaphragm, during the inhalation of chloroform, will cause death promptly.

"The same experiments has found that chloroform would produce fatal consequences wherever the phrenic nerves had been previously tied.

"M. Giraudet declares that, of all the means to which he has resorted in his efforts to restore animals to life, after the inhalation of chloroform, none will compare with the effect produced by the passage of electro-magnetic currents through the diaphragm, or in establishing a current along the course of the phrenic nerves."

